# Vitamin D Deficiency and Long-Term Cognitive Impairment Among Older Adult Emergency Department Patients

**DOI:** 10.5811/westjem.2019.8.43312

**Published:** 2019-10-16

**Authors:** Christopher S. Evans, Wesley Self, Adit A. Ginde, Rameela Chandrasekhar, E. Wesley Ely, Jin H. Han

**Affiliations:** * Vanderbilt University Medical Center, Department of Emergency Medicine, Nashville, Tennessee; † University of Colorado School of Medicine, Department of Emergency Medicine, Aurora, Colorado; ‡ Vanderbilt University, Department of Biostatistics, Nashville, Tennessee; § Vanderbilt University Medical Center, Department of Medicine, Division of Allergy Pulmonary, and Critical Care Medicine, Nashville, Tennessee; ¶ Vanderbilt University Medical Center, Department of Emergency Medicine and Geriatric Research, Education, and Clinical Center, Tennessee Valley Healthcare System, Nashville, Tennessee

## Abstract

**Introduction:**

Approximately 16% of acutely ill older adults develop new, long-term cognitive impairment (LTCI), many of whom initially seek care in the emergency department (ED). Currently, no effective interventions exist to prevent LTCI after an acute illness. Identifying early and modifiable risk factors for LTCI is the first step toward effective therapy. We hypothesized that Vitamin D deficiency at ED presentation was associated with LTCI in older adults.

**Methods:**

This was an observational analysis of a prospective cohort study that enrolled ED patients ≥ 65 years old who were admitted to the hospital for an acute illness. All patients were enrolled within four hours of ED presentation. Serum Vitamin D was measured at enrollment and Vitamin D deficiency was defined as serum concentrations <20 mg/dL. We measured pre-illness and six-month cognition using the short form Informant Questionnaire on Cognitive Decline in the Elderly (IQCODE), which ranges from 1 to 5 (severe cognitive impairment). Multiple linear regression was performed to determine whether Vitamin D deficiency was associated with poorer six-month cognition adjusted for pre-illness IQCODE and other confounders. We incorporated a two-factor interaction into the regression model to determine whether the relationship between Vitamin D deficiency and six-month cognition was modified by pre-illness cognition.

**Results:**

We included a total of 134 older ED patients; the median (interquartile range [IQR]) age was 74 (69, 81) years old, 61 (46%) were female, and 14 (10%) were nonwhite race. The median (IQR) vitamin D level at enrollment was 25 (18, 33) milligrams per deciliter and 41 (31%) of enrolled patients met criteria for vitamin D deficiency. Seventy-seven patients survived and had a six-month IQCODE. In patients with intact pre-illness cognition (IQCODE of 3.13), Vitamin D deficiency was significantly associated with worsening six-month cognition (β-coefficient: 0.43, 95% CI, 0.07 to 0.78, p = 0.02) after adjusting for pre-illness IQCODE and other confounders. Among patients with pre-illness dementia (IQCODE of 4.31), no association with Vitamin D deficiency was observed (β-coefficient: −0.1;, 95% CI, [−0.50–0.27], p = 0.56).

**Conclusion:**

Vitamin D deficiency was associated with poorer six-month cognition in acutely ill older adult ED patients who were cognitively intact at baseline. Future studies should determine whether early Vitamin D repletion in the ED improves cognitive outcomes in acutely ill older patients.

## INTRODUCTION

Long-term cognitive impairment (LTCI), defined as new or worsening deficit in cognition that persists following acute illness, is a well described phenomenon occurring in an estimated 16% of older adults who are acutely ill.^1^ This often leads to increased disability, loss of independence, and decreased quality of life. Currently no effective therapies, especially those that can be administered early in the acute illness course, exist to prevent or treat LTCI following acute illness.

While the mechanism of LTCI has not been fully elucidated, it is hypothesized that systemic proinflammatory cytokines, in response to an acute medical illness such as sepsis,^2^ lead to increased central nervous system (CNS) inflammation, microglial activation, and neuronal injury and death.^2^ Vitamin D is a pleotropic hormone that modulates systemic and CNS inflammatory responses.^3^ Therefore, patients with Vitamin D deficiency may be particularly vulnerable to LTCI following an acute illness. Several observational studies have suggested that Vitamin D deficiency is associated with poorer long-term cognition among community-dwelling adults.^4^ However, the relationship between Vitamin D deficiency in the setting of acute illness and subsequent development of LTCI remains unknown in acutely ill patients, especially in the emergency department (ED) setting. Therefore, we sought to determine whether serum Vitamin D at ED presentation was associated with poorer six-month cognition in acutely ill older adults.

## METHODS

This study was an observational secondary analysis within the DELINEATE prospective cohort study, which enrolled ED patients age 65 years and older who were subsequently admitted the hospital for an acute illness at a large, academic, tertiary care hospital.^5^ This study enrolled patients from March 2012 – November 2014. The local institutional review board reviewed and approved this study.

Details and rationale of the selection of participants have been described previously.^5^ Briefly, we included patients if they were 65 years or older and in the ED for less than four hours at the time of enrollment. Patients were excluded if they were non-English speaking; previously enrolled; deaf, comatose, non-verbal or unable to follow simple commands prior to their current illness; were considered unsuitable for enrollment by the treating physician or nurse; were unavailable for enrollment within the four-hour time limit secondary to clinical care (eg, procedures, radiologic testing, etc,); or were discharged home from the ED. Patients were included for this analysis if they had blood specimen available for Vitamin D measurement and had a surrogate available to complete a short form Informant Questionnaire on Cognitive Decline in the Elderly (IQCODE) obtained at enrollment to establish pre-illness cognition.

Pre-illness (baseline) and six-month cognition (primary outcome) were measured using the short form IQCODE in patients who had a surrogate in the ED who knew the patient for greater than 10 years. It ranges from 1 to 5 (severe cognitive impairment). This surrogate-based cognitive screen was used because patient-based measurements in the ED may not accurately reflect true baseline cognition especially in the setting of delirium.^6^ The IQCODE is also a validated measure of cognition, which has been previously used to assess cognitive decline^5^,^7^ At time of study enrollment, informants were asked to assess the patients’ pre-illness cognition at two weeks prior to ED presentation, and follow-up assessment at six months over telephone with all attempts made to have the same person complete the IQCODE questionnaire as the individual who completed the pre-illness questionnaire.

The primary independent variable was serum Vitamin D measured at ED enrollment. We used Vitamin D level at ED presentation to identify patients with pre-existing Vitamin D deficiency prior to hospitalization for an acute illness. Vitamin D deficiency was defined as a serum Vitamin D concentration <20 milligrams per deciliter (mg/dL).^2^ We collected blood in citrate anti-coagulated collection tubes immediately upon study enrollment. Tubes were placed on ice and centrifuged at 3000 g-force within one hour to isolate plasma. Samples were stored at −80C until batched Vitamin D measurements were performed using the Abbott Architect i2000 (Abbott Pharmaceuticals, Lake Bluff, IL).

We used the Charlson comorbidity index to quantify patient comorbid burden.^8^ The Acute Physiology Score (APS) of the Acute Physiology and Chronic Health Evaluation II (APACHE II) score, including age, was used to quantify severity of illness.^9^ The presence of a CNS diagnosis (meningitis, seizure, cerebrovascular accident, transient ischemic attack, intraparenchymal hemorrhage, epidural hematoma, subdural hematoma, subarachnoid hemorrhage, cerebral edema, meningitis, etc,) was determined by two physician reviewers via medical record review. Any disagreement was adjudicated by a third physician reviewer.

### Statistical Analysis

To determine whether Vitamin D was associated with poorer six-month cognition, we performed multiple linear regression with Vitamin D deficiency as a binary variable adjusting for covariates, IQCODE, the Charlson comorbidity index, APS, and presence of a CNS diagnosis, which were all defined a priori. Because we previously observed that pre-existing cognition may modify any associations and long-term cognition,^10^ we incorporated a cross product of Vitamin D and pre-illness IQCODE in the linear regression model. If the interaction p-value was < 0.25, then it was retained in the multiple regression model. Because the IQCODE is a continuous variable, vitamin D’s b-coefficients are reported at the 25th and 75th percentile values of the pre-illness IQCODE to represent those who were cognitively intact and cognitively impaired at baseline, respectively. Another multivariable model was run where serum Vitamin D deficiency was the independent variable. We used SAS version 9.4 (SAS Institute, Cary, NC) for statistical analysis.

## RESULTS

Of the 228 patients enrolled in the DELINEATE cohort, 30 patients did not have a surrogate present to complete the pre-illness IQCODE and 64 did not have blood collected at enrollment leaving 134 participants available for this analysis. The patient characteristics stratified by Vitamin D deficiency status can be seen in the Table. The median (interquartile range) Vitamin D level at enrollment was 25 (18,33) mg/dL and 41 (31%) patients met criteria for Vitamin D deficiency.^11^

Of the 134 patients, 25 (18.7%) died prior to the six-month follow-up, four (3.0%) opted out of the follow-up at enrollment, 10 (7.5%) were lost to follow-up, and 18 (13.4%) were successfully followed-up but a surrogate was not readily available to complete the six-month IQCODE. A total of 77 patients survived and had a six-month IQCODE. The interaction term between Vitamin D deficiency and pre-illness IQCODE interaction’s p-value was significant (p = 0.0111) indicating that the relationship between Vitamin D deficiency and six-month IQCODE was modified by the pre-illness IQCODE. The Figure displays the multivariable linear regression models between serum Vitamin D at ED enrollment and adjusted six-month cognition. Among patients with a pre-illness IQCODE of 3.13 (cognitively intact at baseline), for every 1 mg/dL decrease in serum Vitamin D, the six-month IQCODE score significantly increased by 0.18 points (95%CI: 0.00 to 0.031) after adjusting for pre-illness IQCODE and other potential confounders; this indicated that lower serum Vitamin D concentrations measured at ED enrollment was associated with poorer six-month cognition. Among those with an IQCODE of 4.313 (cognitively impaired at baseline), no association with Vitamin D deficiency was observed (β-coefficient: 0.00; 95% CI, −0.01 to 0.02). Similarly, Vitamin D deficiency was significantly associated with worsening six-month cognition (adjusted β-coefficient: 0.44; 95% CI, 0.09 to 0.79) among older adults cognitively intact at baseline (pre-illness IQCODE = 3.13). No association with Vitamin D deficiency was seen (β-coefficient: −0.10, 95% CI, −0.49 to 0.29) in those with pre-illness cognitive impairment (pre-illness IQCODE = 4.313).

## DISCUSSION

Our findings suggest that Vitamin D deficiency is common among older patients presenting to the ED with an acute medical illness, and Vitamin D deficiency is associated with increased risk for LTCI among older adults who are cognitively intact prior to an acute illness. Unfortunately, no intervention exists to preserve long-term cognition after an acute illness. The first step toward discovering an intervention is to identify modifiable risk factors early on in the course of an acute illness, and this is the impetus for our study. Future studies should determine if early Vitamin D repletion in the ED improves cognitive outcomes in acutely ill older patients.

We also observed that the association between serum Vitamin D concentrations and six-month cognition was more prominent in patients with intact cognition at baseline. It is possible that Vitamin D deficiency in the setting of acute illness may more profoundly affect those with intact cognition. It is also possible that patients with intact cognition at baseline are more at risk for cognitive decline following acute illness that is detectable with the measures currently available to assess cognition. Future studies should confirm this finding using more robust neuropsychiatric evaluations to quantify long-term cognition.

Our study builds upon the work conducted in the outpatient settings, which also reported that low- serum Vitamin D level is associated with the development of Alzheimer’s disease.^4^ Because systemic and CNS inflammation are the underpinning of LTCI pathophysiology, we hypothesize that Vitamin D treatment could potentially improve long-term cognition by attenuating systemic and CNS inflammatory responses. Vitamin D is a pleiotropic secosteroid hormone that modulates systemic and CNS inflammatory responses.^12^ Inflammation in response to an acute illness plays a prominent role in LTCI pathogenesis.^13^ Vitamin D down-regulates systemic inflammation by inhibiting the release of peripheral pro-inflammatory cytokines such as tumor necrosis factor-α (TNF-α), IL-6 and IL-12.^14^,^15^ Additionally, Vitamin D also inhibits CNS inflammation by attenuating systemic inflammation and more directly by specifically targeting the brain. Based upon in-vitro models, Vitamin D further attenuates CNS inflammation by inhibiting microglial production of pro-inflammatory cytokines such as IL-6 and TNF-α.^14^

## LIMITATIONS

Our study has several limitations. First, the study was observational and thus was only able to assess association, not causation, between Vitamin D deficiency and long-term cognitive impairment. We used IQCODE to measure pre-illness and six-month cognition. While it has been previously used to characterize cognitive decline, it is possible that misclassification could have occurred and may have biased our findings.

Second, selection bias may have been introduced by enrolling patients from a single site and only during weekdays and daytime hours. Our primary analysis was based on 57% of enrolled patients due to death during the study period or no six-month IQCODE available for analysis. Patients with missing six-month IQCODEs were similar in age, pre-illness OARS ADL, comorbidity burden, severity of illness, proportion with altered mental status as a chief complaint, and the proportion with CNS diagnosis compared those with accessible six-month IQCODEs (Supplemental Table). However, patients with missing six-month IQCODEs were more likely to be female, non-white race, and cognitively intact at baseline.

Furthermore, our study was limited to a single, academic, tertiary care hospital; thus, our findings may not be generalizable to other settings. Inherent to observational studies and limited by our sample size, unmeasured and residual confounding may exist.

## CONCLUSION

In older adults without pre-existing cognitive impairment (eg, dementia), Vitamin D deficiency at ED presentation was a risk factor for poorer six-month cognition. This relationship was present after adjusting for confounders chosen a priori such as other comorbidities, severity of illness, and the presence of pre-existing cognitive impairment and other CNS diagnoses. We found no such association with Vitamin D and LTCI among older adults with pre-illness cognitive impairment. This suggests that vitamin D deficiency among cognitively intact older adults at the time of ED presentation may be a potentially modifiable risk factor in the development of long-term cognitive impairment.

## 

## Figures and Tables

**Figure:**
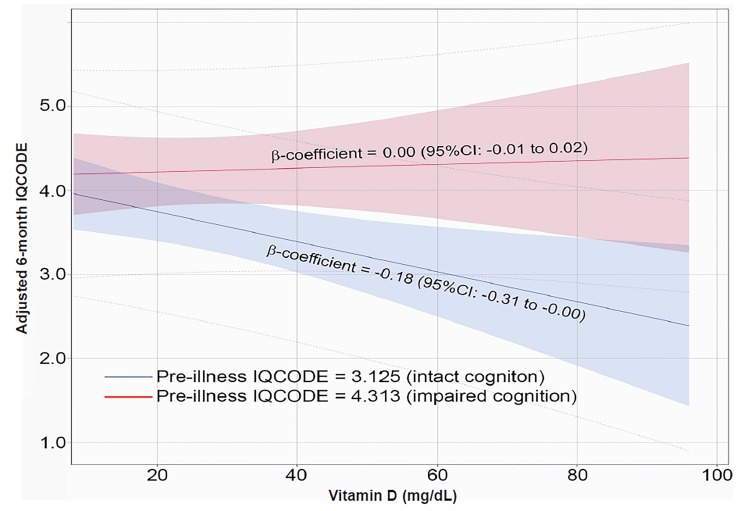
The relationship between serum Vitamin D concentrations measured at enrollment and 6-month cognition. Cognition was determined by the short Informant Questionnaire on Cognitive Decline in the Elderly (IQCODE) which ranged from 1 to 5 (severe cognitive impairment). The association between serum Vitamin D and 6-month cognition was modified by pre-illness cognition. In older adults with pre-illness cognitive impairment (higher IQCODE group, shown in RED), serum Vitamin D concentrations were not a predictor of adjusted 6-month cognition. In older adults who were cognitively intact at baseline (lower pre-illness IQCODE group, shown in BLUE), there was a statistically significant relationship between serum vitamin D concentrations and 6-month cognition after adjusting for confounders.

**Table:** Patient characteristics and demographics.

	Non-Vitamin D Deficient*	Vitamin D Deficient*
Median age (IQR)	74 (69, 82)	72 (67, 79)
Female gender	51 (54.8%)	22 (53.7%)
Non-white race	6 (6.5%)	8 (19.5%)
Median pre-illness IQCODE (IQR)	3.56 (3.06, 4.13)	3.19 (3.00, 4.00)
Median OARS ADL (IQR)	22 (15, 27)	25 (17, 27)
Median Charlson Comorbidity Index (IQR)	4 (3, 6)	4 (2, 5)
Median APS (IQR)	13 (12, 15)	15 (13, 18)
CNS diagnosis	20 (21.5%)	3 (7.3%)
ED chief complaint		
Abdominal pain	4 (4.4%)	4 (10.3%)
Altered mental status	16 (17.4%)	7 (18.0%)
Chest pain	6 (6.5%)	5 (12.8%)
Generalized weakness	9 (9.8%)	2 (5.1%)
Nausea/vomiting	5 (5.4%)	0 (0.0%)
Shortness of breath	10 (10.9%)	5 (12.8%)
Syncope	4 (4.4%)	0 (0.0%)

*IQR*, Interquartile range; *APS*, Acute Physiology Score; *IQCODE*, Informant Questionnaire on Cognitive Decline in the Elderly score; *OARS ADL*, Older American Services Activities of Daily Living; *CNS*, central nervous system.

The APS also incorporates age from the Acute Physiology and Chronic Health Evaluation II (APACHE II).

* Vitamin D deficiency was defined as a serum concentration of less than 20 ng/dL.
