# Supplementary material for: Vitamin D Deficiency and Long-Term Cognitive Impairment Among Older Adult Emergency Department Patients
Source: West J Emerg Med. 2019 Oct 16;20(6):926–30. doi: 10.5811/westjem.2019.8.43312 (PMC6860383; doi:10.5811/westjem.2019.8.43312)
Supplement: Supplementary file 1 [file wjem-20-926-s001.docx]

**Supplemental Table:** Patient characteristics and demographics stratified by missing 6-month IQCODE.

|  | **Non-Missing**  **6-month IQCODE**  **n=77** | **Missing**  **6-month IQCODE**  **n=57** |  |  |
| --- | --- | --- | --- | --- |
| Vitamin D Deficiency | 22 (28.6%) | 19 (33.3%) |  |  |
| Median Age (IQR) | 73 (67, 79) | 74 (70, 82) |  |  |
| Female gender | 37 (48.1%) | 36 (63.2%) |  |  |
| Non-white race | 6 (7.8%) | 8 (14.0) |  |  |
| Median IQCODE (IQR) | 3.56 (3.06, 4.50) | 3.31 (3.0, 3.75) |  |  |
| Median OARS ADL (IQR) | 23 (16, 27) | 22 (15, 27) |  |  |
| Median Charlson Comorbidity Index (IQR) | 2 (1, 4) | 3 (2, 6) |  |  |
| Median APS (IQR) | 13 (12, 15) | 14 (12, 16) |  |  |
| CNS Diagnosis | 13 (16.8%) | 10 (17.5%) |  |  |
| ED Chief Complaint  Abdominal pain  Altered mental status  Chest pain  Generalized weakness  Nausea / vomiting  Shortness of breath  Syncope | 6 (7.8%)  13 (16.9%)  6 (7.8%)  5 (6.5%)  3 (3.9%)  6 (7.8%)  2 (3.6%) | 2 (3.5%)  10 (17.5%)  5 (8.8%)  6 (10.5%)  2 (3.5%)  9 (15.8%)  2 (3.5%) |  |  |

*IQR*, Interquartile range; *APS*, Acute Physiology Score; *IQCODE*, Informant Questionnaire on Cognitive Decline in the Elderly score; *OARS ADL*, Older American Services Activities of Daily Living; *CNS*, central nervous system.

The APS also incorporates age from the Acute Physiology and Chronic Health Evaluation II (APACHE II).
